# *Cryptococcus gattii* Virulence Composite*:* Candidate Genes Revealed by Microarray Analysis of High and Less Virulent Vancouver Island Outbreak Strains

**DOI:** 10.1371/journal.pone.0016076

**Published:** 2011-01-13

**Authors:** Popchai Ngamskulrungroj, Jennifer Price, Tania Sorrell, John R. Perfect, Wieland Meyer

**Affiliations:** 1 Molecular Mycology Research Laboratory, Centre for Infectious Diseases and Microbiology, Sydney Medical School - Westmead, Westmead Millennium Institute, Sydney Emerging Infections and Biosecurity Institute, The University of Sydney, Westmead Hospital, Westmead, New South Wales, Australia; 2 Department of Medicine, Duke University Medical Center, Durham, North Carolina, United States of America; 3 Faculty of Medicine Siriraj Hospital, Mahidol University, Bangkok, Thailand; Research Institute for Children and the Louisiana State University Health Sciences Center Pediatrics, United States of America

## Abstract

Human and animal cryptococcosis due to an unusual molecular type of *Cryptococcus gattii* (VGII) emerged recently on Vancouver Island, Canada. Unlike *C. neoformans, C. gattii* causes disease mainly in immunocompetent hosts, despite producing a similar suite of virulence determinants. To investigate a potential relationship between the regulation of expression of a virulence gene composite and virulence, we took advantage of two subtypes of VGII (a and b), one highly virulent (R265) and one less virulent (R272), that were identified from the Vancouver outbreak. By expression microarray analysis, 202 genes showed at least a 2-fold difference in expression with 108 being up- and 94 being down-regulated in strain R265 compared with strain R272. Specifically, expression levels of genes encoding putative virulence factors (e.g. *LAC1, LAC2, CAS3* and *MPK1*) and genes encoding proteins involved in cell wall assembly, carbohydrate and lipid metabolism were increased in strain R265, whereas genes involved in the regulation of mitosis and ergosterol biosynthesis were suppressed. *In vitro* phenotypic studies and transcription analysis confirmed the microarray results. Gene disruption of *LAC1* and *MPK1* revealed defects in melanin synthesis and cell wall integrity, respectively, where *CAS3* was not essential for capsule production. Moreover, *MPK1* also controls melanin and capsule production and causes a severe attenuation of the virulence in a murine inhalational model. Overall, this study provides the basis for further genetic studies to characterize the differences in the virulence composite of strains with minor evolutionary divergences in gene expression in the primary pathogen *C. gattii,* that have led to a major invasive fungal infection outbreak.

## Introduction

The primary pathogen *Cryptococcus gattii,* previously designated as *Cryptococcus bacillisporus* or *Cryptococcus neoformans* var. *gattii,* is a basidiomycetous yeast that in Australia causes disease primarily in immunocompetent hosts [1]. It is a member of the *Cryptococcus neoformans/C. gattii* species complex [2]. Until recently, *C. gattii* was considered to be a tropical and subtropical pathogen [3]. However, the emergence of cryptococcosis due to an uncommon molecular type of *C. gattii* (VGII) in humans and animals on Vancouver Island, BC, Canada, and its more recent spread to mainland British Columbia and the adjacent Pacific Northwest of the United States has changed this paradigm [4], [5], [6], [7], [8], [9]. In addition, *C. gattii* has caused human disease in the temperate climates of France, Italy, Spain, Greece and Colombia [10], [11], [12], [13], [14].

The Vancouver Island outbreak emphasized the importance of understanding the pathobiology of this emerging pathogen [6]. An early study revealed that a strain (R265) of the predominant subtype in the outbreak (VGIIa), was more virulent for A/Jcr mice than an isolate (R272) of the minor subtype (VGIIb), the proposed parental strain of R265 [15]. Subsequently it was shown in a sample of 40 VGIIa and VGIIb strains from around the world that the VGIIa subtype produced more of the virulence determinant, melanin, than did the VGIIb subtype [16]. The increased virulence of the VGIIa subtype was confirmed investigating 21 globally-collected *C. gattii* strains in Balb/c mice [17]. Approximately one-half of the gene sequences used as MLST markers in the R265 and R272 strains are identical [15].

Since the beginning of the AIDS era, several cryptococcal virulence factors have been identified using *C. neoformans* as a model [18]. Melanin [19], capsule [20], growth at 37°C [21], mating type α [22], phospholipase [23], superoxide dismutases [24], protein kinases [25], urease [26] and cell wall integrity enzymes [27] have been phenotypically and molecularly characterized as virulence factors that drive invasion of the host and survival *in vivo* [18]. Few studies have been performed in *C. gattii* and hence the genes regulating its virulence composite have been inferred from the work in *C. neoformans*. Recently some evidence for differences in virulence gene regulation between these two sibling species has been published [28], [29].

Only a limited number of virulence genes have been characterized in *C. gattii*, namely phospholipase B [30], superoxide dismutases [31], protein kinases [28], a transcriptional factor [32] and the trehalose biosynthetic pathway [29]. The substantial outbreak of cryptococcosis due to *C. gattii* in the south-west of Canada and the north-west of the US has focused attention on the importance of *C. gattii* as a primary and emerging pathogen [6]. The study of the initial *C. gattii* strains from the Vancouver Island outbreak suggests that following a natural event involving recombinational exchange between two ancestral strains, progeny were produced, which were much more virulent than the parental strains [15]. Taking advantage of this natural experiment in pathobiology, we utilized the high and lower virulence strains in an attempt to identify key components of the *C. gattii* virulence composite. A recent whole-genome expression study identified mitochondrial regulation as an important factor in the enhanced virulence of strain R265 [33]. Another revealed that suppression of the host immune response is pivotal to the virulence of this yeast [34]. However, neither of these studies involved characterization of the responsible genes. Thus, we conducted a comparative transcriptional study between a representative highly virulent VGIIa strain, R265, and a representative lower virulent VGIIb strain, R272, which have been used extensively in previous genetic and virulence studies [6], [15], [35]. Numerous differences in gene regulation were indentified. Differentially expressed genes potentially responsible for the virulence composite of *C. gattii* were further characterized, and their importance was confirmed by phenotypic analyses of virulence.

## Results

### Microarray analysis

The microarray analysis was performed under conditions of carbon and nitrogen starvation and growth at 37°C [36], to mimic stress conditions as they might be encountered during a human CSF infection. Extensive transcriptional differences between the high and low virulent strains, R265 and R272, respectively were identified (Table S1). All raw data from triplicate experiments have been submitted to MIAME (http://www.ebi.ac.uk) under the ArrayExpress accession number: A-MEXP-1133.

Table S2 lists the gene functions of the differentially expressed genes identified in the microarray experiments via BLASTN searches against the GenBank database. The genes are grouped according to their putative biological functions. Open reading frames with unknown functions were excluded, but can be found in the Table S1. To follow up a previous reported connection between *C. gattii* virulence and mitochondrial gene expression [33], a blast search against the draft R265 genome was performed. However, no specific correlation with the mitochondrial genome, located on the supercontig 25, was found (Table S2).

Among the up-regulated genes, there were several genes which are related to the three major virulence factors of *C. neoformans,* which include: 1) melanin: *LAC1* and *LAC2* [19], [37] and the copper chaperone, *ATX1* [38]; 2) capsule: the capsule-associated gene, *CAS3* [39] and 3) growth at 37°C: the MAP kinase signaling gene, *MPK1* which is critically important for the growth of *C. neoformans* at 37°C [21] (Table S2).

*MPK1* is also known to regulate cell integrity by controlling the expression of several genes involved in cell wall assembly [40]. We noted that genes involved in cell wall assembly in *S. cerevisiae* [41] were also differentially expressed in strain R265, including glucan/chitin biosynthesis and remodeling genes: 1) β-1,6 glucan synthetase, [42], 2) glucanases [43], 3) chitin synthases, [41], 4) chitin deacetylase [44] and 5) UDP-N-acetylglucosamine diphosphorylase [43]. In contrast, a chitinase gene (*CNI0386*), which plays a role in cell separation during budding [43] was down-regulated (Table S2).

Many genes involved in cell metabolism, including carbohydrate, lipid and ergosterol metabolism, were down-regulated in the strain R265. Genes involved in cell division and cryptococcal pheromone receptors (*CPRα/STE3*) [45] were also down-regulated, possibly to maintain microbial fitness (Table S2).

### Transcriptional analysis of selected up- and down-regulated genes

Transcription of differentially-expressed genes was confirmed by real time PCR. Transcription levels of *LAC1, CAS3, LAC2, MPK1* and *STE3* and *ETF1α* genes identified by RT-PCR correlated with expression levels identified in the microarray experiments (see footnote to Table S2).

### Phenotypic comparisons between the strain R265 and R272

Since several potential virulence genes namely laccase, a capsule-associated gene, MAP kinase, cryptococcal pheromone receptor and ergosterol biosynthesis genes were differentially expressed, melanin synthesis, capsule production, cell wall integrity/growth at 37°C, fertility and azole sensitivity were compared.

### Melanin synthesis

**Figure 1:**
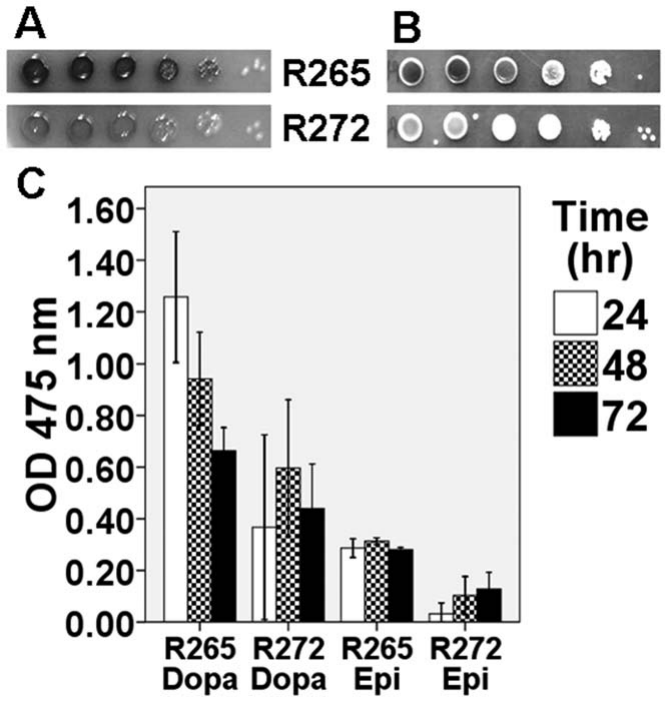
Melanin production of strains R265 and R272. Strain R265 produced more melanin on all melanin inducing media, e.g. **A**) bird seed agar at 48 hr at 30°C and **B**) caffeic acid agar at 48 h at 37°C. **C**) Quantitative measurement of the melanin production shows a higher laccase activity for strain R265 with either dopamine (Dopa) or epinephrine (Epi) as substrates (standard error bar +/− 2SE).

Strain R265 produced more melanin than strain R272. Specifically, at 30°C, melanin production in strain R265 was relatively increased on all three melanin-inducing media (Niger seed agar, dopamine agar and caffeic acid agar) and even more so at 37°C. This was confirmed by quantification of the laccase activity on two substrates, dopamine and epinephrine, (p = 0.004) (Figure 1).

### Capsule production

**Figure 2:**
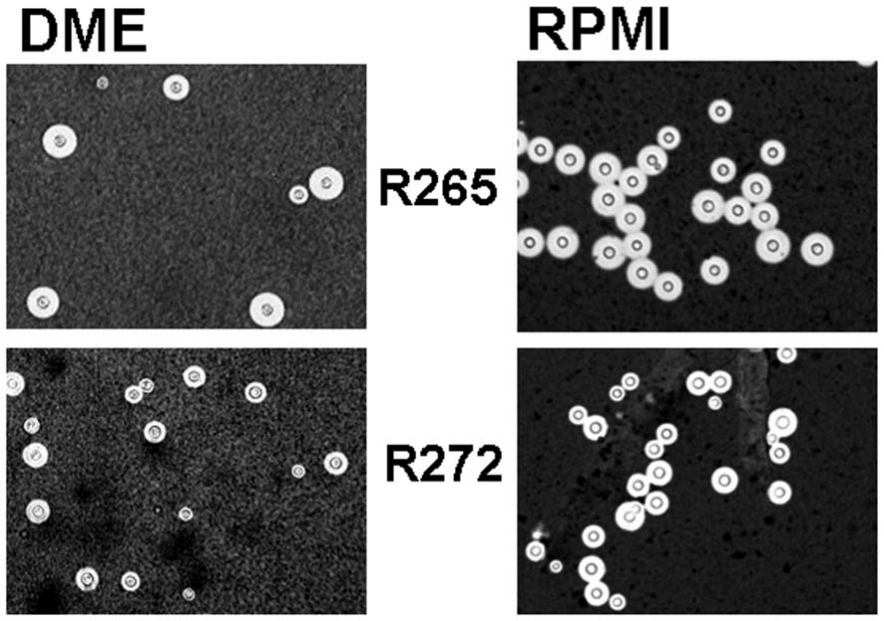
Capsule formation of strains R265 and R272. Yeast cells were grown in DME at 37°C with 5% CO_2_ and RPMI-1640 medium at 37°C with 5% CO_2_ for 72 h.

When cultured in three different capsule-inducing conditions, capsule size was larger in strain R265 than in strain R272. The average capsule to capsule: cell wall to cell wall ratios of strain R265 compared to strain R272 were 2.1 versus 1.8 in DME and 3.0 versus 2.1 in RPMI with CO_2_ (Figure 2). All results were statistically significant (p = 0.005 for DME and p<0.001 for RPMI).

### Cell wall integrity and growth at 37°C

**Figure 3:**
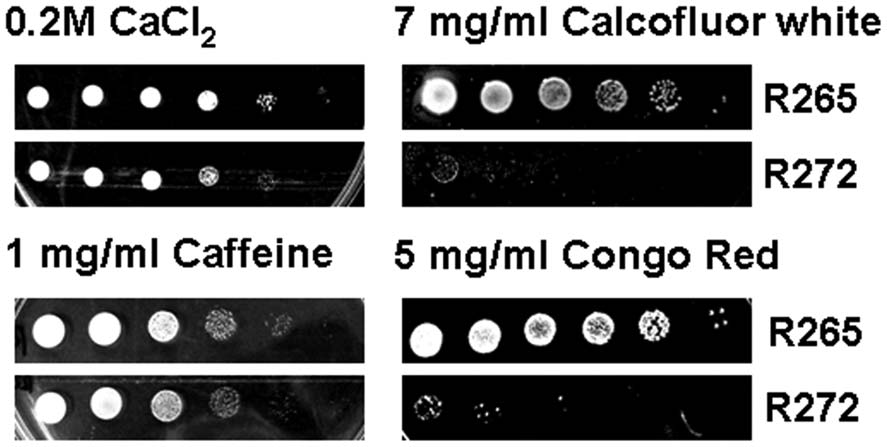
Cell wall integrity tests for strain R265 vs. strain R272 isolates after incubation in YPD medium supplemented with CaCl_2,_ calcofluor white (CFW), caffeine and Congo red.

R265 was more tolerant than R272 to cell wall perturbing agents calcofluor white and congo red, and slightly more tolerant to CaCl_2_ and caffeine (Figure 3). On the other hand, although the cell wall integrity pathway is known to be linked to high temperature tolerance, the growth rate of the strain R272 at 37°C was reduced in minimal and not highly nutritious media (data not shown).

### Fertility

**Figure 4:**
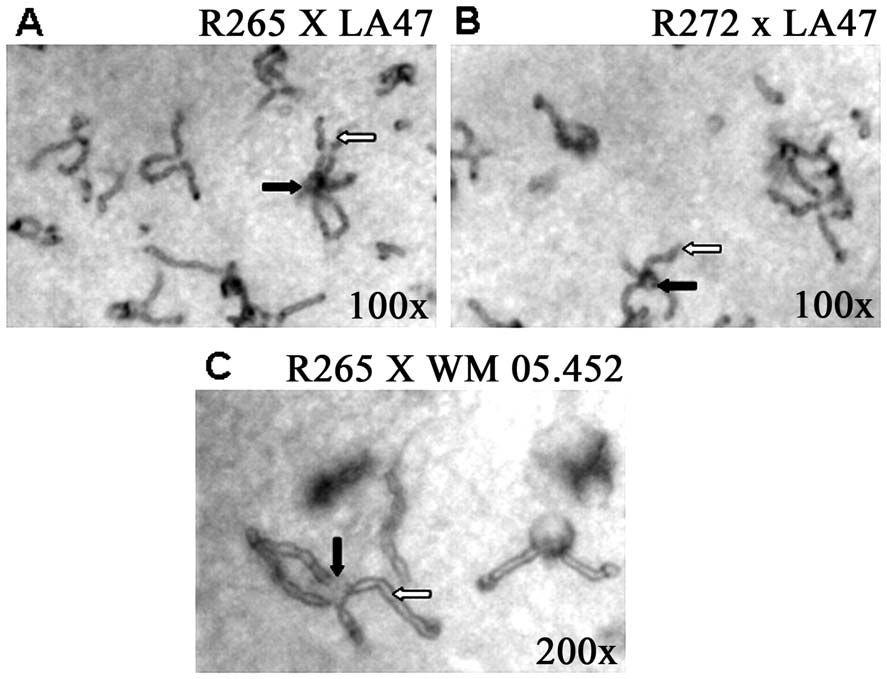
Mating reactions of the Vancouver Island isolates showing similar mating properties, with typical bacilli shaped basidiospores being observed for both strains [**72**]. The dark arrows present basidia and the white arrows identify basidiospores.

R265 was more fertile than R272, mating with 14 (78%) of the 18 VGII MAT**a** strains tested, while strain R272 mated only with 9 (50%) of the 18 tested strains (p = 0.083) (Table 1). The mating structures were typical of VGII hyphae, basidia and basidiospores as described in a previous study (Figure 4) [46].

**Table 1:** List of strains used in this study.

Name	Country	Source	Mating type	Sub-type	Fertility when mating with		Reference
					R265	R272	
R265	Canada	Clinical	α	VGIIa	N/A	N/A	[6]
R272	Canada	Clinical	α	VGIIb	N/A	N/A	[6]
R265*lac1Δ*	N/A	N/A	α	N/A	N/A	N/A	This study
R265*cas3Δ*	N/A	N/A	α	N/A	N/A	N/A	This study
R265*mpk1Δ*	N/A	N/A	α	N/A	N/A	N/A	This study
R265*lac1Δ::LAC1*	N/A	N/A	α	N/A	N/A	N/A	This study
R265*cas3Δ::CAS3*	N/A	N/A	α	N/A	N/A	N/A	This study
R265*mpk1Δ::MPK1*	N/A	N/A	α	N/A	N/A	N/A	This study
LA 47	Brazil	Clinical	a	N/A	F	F	[12]
LA 55	Brazil	Clinical	a	N/A	F	F	[12]
LA 381	Venezuela	Clinical	a	N/A	N	N	[12]
LA 461	Colombia	Clinical	a	N/A	N	N	[12]
LA 499	Colombia	Clinical	a	N/A	F	N	[12]
LA 516	Colombia	Clinical	a	N/A	F	F	[12]
LA 517	Colombia	Clinical	a	N/A	F	F	[12]
LA 524	Colombia	Clinical	a	N/A	F	N	[12]
LA 540	Colombia	Clinical	a	N/A	F	F	[12]
LA 543	Colombia	Clinical	a	N/A	N	N	[12]
LA 547	Colombia	Clinical	a	N/A	F	N	[12]
LA 567	Colombia	Clinical	a	N/A	F	N	[12]
LA 584	Colombia	Clinical	a	N/A	F	F	[12]
LA 599	Colombia	Clinical	a	N/A	N	N	[12]
CBS1930	Aruba	Veterinary	a	N/A	F	F	[73]
WM 05.452	Brazil	Clinical	a	N/A	F	F	[74]
WM 05.415	Brazil	Clinical	a	N/A	F	F	[74]
AV55	Greece	Cilnical	a	N/A	F	N	[13]

N/A =  not applicable, N  =  infertile, F  =  fertile.

### Susceptibility to the antifungal drug, fluconazole

Fluconazole inhibits C14 α-demethylase (*ERG11*) [47], an enzyme in the ergosterol biosynthetic pathway. Azole susceptibility was tested since several genes involved in ergosterol synthesis were differentially expressed between the strains R265 and R272 (Table S2). The minimal inhibitory concentration of fluconazole for both strains was 4 µg/ml.

### Characterization of the *lac1Δ*, *cas3Δ* and *mpk1Δ* mutants in *C. gattii*

As strain R265 showed higher melanin synthesis, capsule production and higher cell wall integrity/growth at 37°C, the highly expressed genes (Table S2) potentially responsible for these major virulence factors were characterized in the high virulence strain, R265. The *LAC1* (BROAD Institute gene accession No. CNBG_2144.2), *CAS3* (BROAD Institute gene accession No. CNBG_0047.2) and *MPK1* (BROAD Institute gene accession No. CNBG_2556.2) genes in strain R265 were isolated as putative 2543-bp, 2121-bp and 1791-bp open reading frames; these had approximately 82%, 84% and 87% similarities to the *C. neoformans* strain JEC21, respectively. Moreover, the coding sequences of the *LAC1*, *CAS3* and *MPK1* genes had approximately 84%, 84% and 92% similarities to the JEC21 genes, respectively (Figure S1).

**Figure 5:**
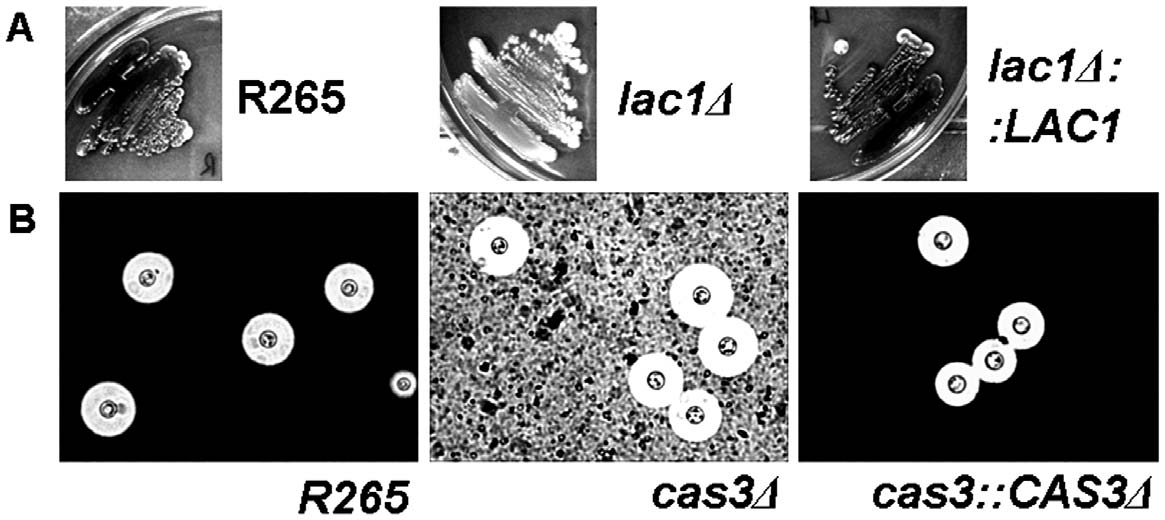
Phenotypic study of *lac1Δ* and *cas3Δ* mutants. A) The *lac1Δ* mutant strain exhibited lower melanin production on caffeic acid agar at 30°C as compared with the wild-type R265 strain and its reconstituted strain (*lac1Δ::LAC1)*. B) No difference in capsule formation was observed in the *cas3Δ* mutant on 5% CO_2_ DME at 37°C as compared to the wild-type R265 strain and the reconstituted strain (*cas3Δ:: CAS3Δ*).

Phenotypic analysis of the *lac1Δ* mutant in strain R265 revealed a severe melanin production defect on all melanin-inducing media (Figure 5A). The complementation of the *LAC1* gene to the *lac1Δ* mutant restored melanin production (Figure 5A). However, the absence of the *CAS3* gene did not affect capsule size in any capsule-induction media (Figure 5B); these results are consistent with previous reports for both *LAC1* and *CAS3* in *C. neoformans* [19], [39]. No other phenotypic defects were found in either *lac1Δ* or *cas3Δ* mutants except melanin deficiency in the *laclΔ* mutant.

**Figure 6:**
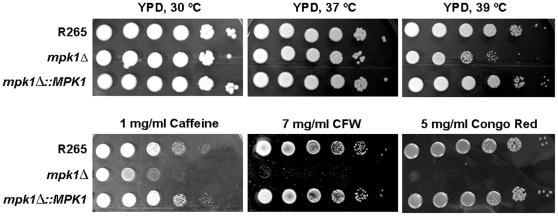
Growth of the wild type R265 stain, *mpklΔ* mutant and reconstituted *mpk1Δ::MPK1* strain under different temperatures and cell wall stress conditions. Cells were grown on YPD at different temperatures or in the presence of cell wall disturbing agents as indicated.

**Figure 7:**
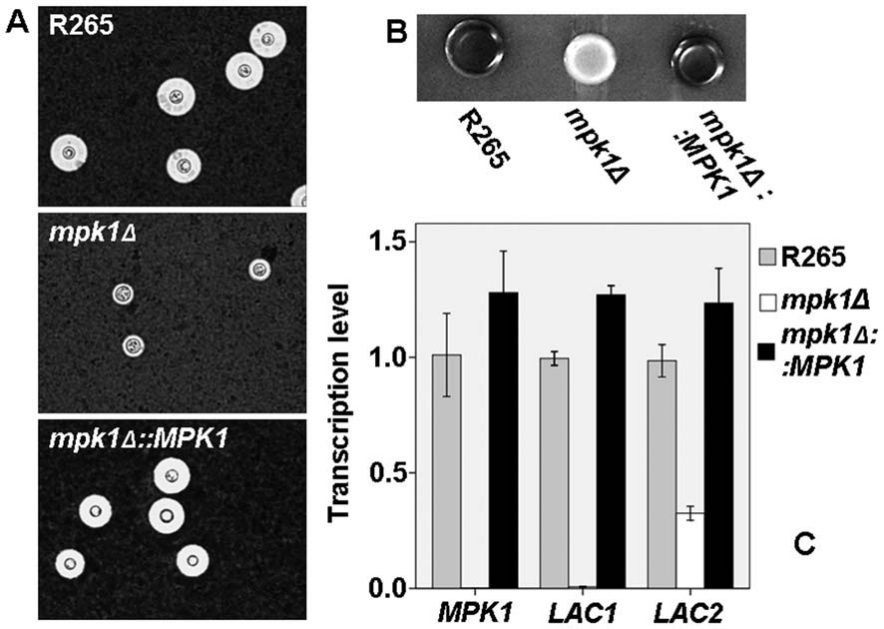
A) Phenotypic impact of *mpklΔ* on capsule, melanin, and control of laccase gene transcription. Capsule production test in DME with 5%CO_2_ at 37°C, B) Melanin synthesis test on Caffeic acid agar at 37°C, C) *MPK1*, *LAC1* and *LAC2* transcription level of the *mpk1Δ* mutant (white bars) were 0, 0.07 and 0.33 times to the wild type R265 strain, respectively. No significant differences between the transcriptions between the wild type and the *mpk1Δ::MPK1* strain.

The R265 *mpk1Δ* mutant showed a mild growth defect at 37°C, which was more obvious at 39°C. Compared to the wild type strain R265 and the R265 *mpk1Δ::MPK1* complemented strain, the sensitivity to cell wall stresses of the *mpk1Δ* mutant was increased in response to caffeine, calcofluor white and congo red (Figure 6). Moreover, the *mpk1Δ* mutant of strain R265 was defective in melanin synthesis and capsule production (Figure 7A and 7B). On the other hand, no defect in filamentation and sporulation was observed in unilateral crossings with designated strains of the opposite mating type (results not shown).

**Figure 8:**
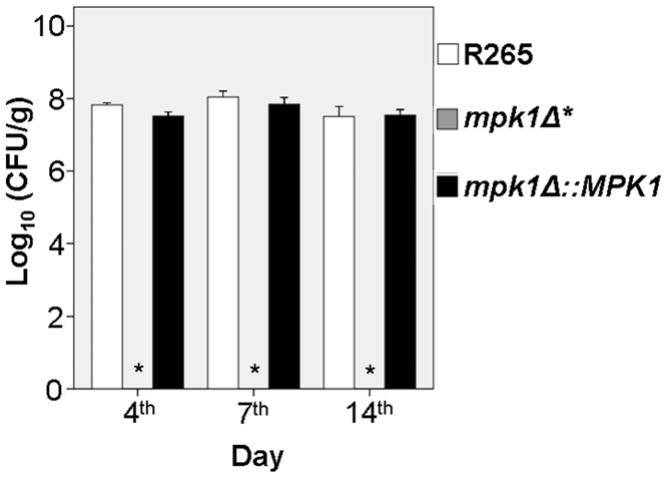
The organ burden of the wild type R265 strain, the *mpk1Δ* mutant and the complemented strain in Balb/c mice lungs. The *mpk1* mutant was not detected in any of the lungs (*).

To test whether melanin production was controlled by *MPK1* via laccases, RT-PCR for transcription of *LAC1* and *LAC2* was performed. *LAC1* and *LAC2* transcription was significantly decreased in the *mpk1Δ* mutant, to 0.07 and 0.33 that of the wild type strain R265 and the complemented strain, respectively (p<0.001 and p = 0.003). Virulence of the *mpk1Δ* mutant was also tested in a murine inhalational model. As expected from the combination of defects in several major virulence factors, the *mpk1Δ* mutants were completely eliminated from the murine lungs by day 4 of the infection (Figure 8).

## Discussion

The present whole genome microarray study revealed extensive differences in the transcriptional profiles of representative high and low virulence strains of *C. gattii* from the Vancouver Island outbreak, the most important of which were confirmed by phenotypic analysis. Genes proven to contribute to the virulence composite of the sibling species, *C. neoformans,* including melanin, capsule, cell wall assembly and mating/spore formation were over-expressed in the highly virulent strain R265. The microarray results were confirmed by RT-PCR and *in vitro* virulence phenotypic studies. Specifically, melanin production, capsule size, cell wall integrity and fertility were higher in strain R265 than in the low virulence strain R272. On the other hand, genes involved in cell cycle and organization of cellular organelles, lipid metabolism and sterol synthesis were down-regulated in strain R265. These findings suggest that either these down-regulated genes are not involved in virulence expression directly or that the level of suppression is not sufficient to reduce the virulence of strain R265. Notably, there was no difference in susceptibility to fluconazole, which inhibits fungal growth primarily by binding to the *ERG11* gene product in the ergosterol biosynthetic pathway, even though the ergosterol biosynthetic genes were down-regulated in strain R265. No specific correlation between virulence and mitochondrial gene expression (R265 supercontig 25) was found in the current study (Table S2), contradicting a previous study, which linked mitochondrial gene regulation with high virulence of the Vancouver Island Outbreak strains when they where compared with isolates from elsewhere [33]. This opposite results are most likely due to the fact that both Vancouver Island Outbreak isolates, R265 and R272, studied in the current investigation, may share a similar mitochondrial type [33] despite their different levels of virulence. As such the results obtained in the current study identify specific expression differences responsible for the differences in the virulence of the two Vancouver Island Outbreak sub-genotypes, VGIIa and VGIIb.

Three classical virulence factors, melanin, capsule and the ability to grow at 37°C, have been described for *C. neoformans* [18]. Many genes have now been linked to these virulence factors. Though several melanin- and capsule-related genes were up-regulated in the high virulent strain R265, regulation or functions of homologous genes may differ between *C. neoformans* and *C. gattii* [28], [29]. To confirm that genes encoding virulence determinants in *C. neoformans,* share a similar role in the virulence composite in *C. gattii,* we created mutants of *LAC1*, *CAS3* and *MPK1* in *C. gattii* using site-directed mutagenesis. Previously established protocols were employed [8]. As expected, *LAC1* and *MPK1* controlled melanin synthesis and cell wall integrity respectively in *C. gattii*. However, although the *CAS3* capsular gene was highly up-regulated in strain R265 compared to strain R272, capsule size was not reduced in the *cas3Δ* mutant. Based on studies in *C. neoformans CAS3* and its gene family are associated with the construction of the polysaccharide capsule backbone [39], but as we observed in *C. gattii, CAS3* does not influence capsule size [39]. We chose to further characterize the *mpk1Δ* mutant since cross talk between the protein kinase C (PKC) signaling pathway and other virulence pathways, especially those involving melanin has been demonstrated in *C. neoformans* [27]. In fact, we found that *MPK1* controlled both melanin and capsule production in strain R265. Further transcription analysis revealed that *MPK1* regulated melanin production through the *LAC1* gene. On the other hand, the essential capsule-producing genes, *CAP10*, *CAP59*, *CAP60* and *CAP64*, were not differentially regulated in the *mpk1Δ* mutant (data not shown). Finally, as a result of the combined defects in the major virulence phenotypes, this mutant exhibited a severe virulence defect measured as survival in our murine inhalational model of *C. gattii* infection.

Whether basidiospores are responsible for human cryptococcosis is still uncertain. However, it has been proposed that basidiospores are the source of infection, as their size are small enough to reach the lung alveoli [48]. Thus, the relative efficiency and density of spore production by either sexual reproduction or haploid fruiting may affect the environmental concentration of infectious particles and hence pathogenicity. A previous study revealed that the Vancouver Island outbreak strains had an unusually high fertility and high concentrations of airborne propagules [6], [8]. When tested for their fertility, strain R265 was more fertile than strain R272 suggesting that the genes controlling mating/sporulation might be more easily induced. Consistent with this hypothesis, the *CHS5* and *CHS7* genes involved in mating were induced in strain R265 but the a-factor pheromone receptor *STE3α* was suppressed. In accordance with the fact that a *C. neoformans CPRα* (*STE3α*) mutant exhibited a defect in sexual filamentation but not haploid fruiting [45], our expression data may favor the occurrence of asexual sporulation in strain R265. On the other hand, glyoxal oxidase genes which are required to determine the mating fertility were in addition to the *CHS5* and *CHS7* genes also up-regulated in strain R265, supporting the higher fertility seen in strain R265. Similar findings were observed in *Ustilago maydis* were the glyoxal oxidase 1, *GLO1*, was also required for filamentous growth and virulence [49].

Genes controlling the cryptococcal cell cycle and organization of cellular organelles may also affect virulence as seen in several fungal pathogen including *U. maydis* [50], *C. neoformans* [51] and *S. cerevisiae* [52]. Thus, the mitotic regulatory genes seem to be important for the fitness of the yeast. Unexpectedly, we found that these genes were suppressed in the more virulent strain R265 of *C. gattii*. However, in *U. maydis*, expression of Clb2 cyclin varies with the stage of infection and is suppressed at distinct stages in the process [53]. A similar scenario might take place in *C. gattii*, which could explain the reduction in the transcription of cell cycle-associated genes in strain R265.

In *C. neoformans*, lipids provide energy and act as regulator(s) of pathobiological processes [54]. Our microarray analysis showed that transcription levels of genes involved in fatty acid β-oxidation differ between the two *C. gattii* strains. Fatty acid β-oxidation has been correlated with the virulence of *U. maydis* [55]. The *MFE2* gene, which encodes a multifunctional enzyme that catalyzes the second and third reactions in β-oxidation of fatty acids in peroxisomes, was necessary for full virulence although it was not required for the production of filaments during mating *in vitro*. In *C. neoformans*, the fatty acid synthase genes (*FAS1* and *FAS2*) are essential [56], but the peroxisomes which contain the enzymes of β-oxidation are not [57]. Although it is uncertain whether this pathway is part of the virulence of *C. gattii*, our expression analysis suggests that it is at least differentially regulated.

Sterols are essential structural and regulatory components of the eukaryotic cell membrane [58]. Ergosterol, an end-product of this biosynthetic pathway, is the main sterol in yeasts. Like cholesterol in mammalian cells, ergosterol affects membrane fluidity and permeability [59]. Although a direct relationship between the ergosterol biosynthetic pathway and cryptococcal virulence has not yet been reported, genes involved in ergosterol synthesis were suppressed in the high virulent strain R265. However, a correlation between virulence and the ergosterol synthesis pathway has been suggested previously [60]. It was reported that a pre-treatment with subinhibitory concentrations of azoles *in vitro* reduced cryptococcal virulence *in vivo* [61]. However, no difference in the susceptibility to the azole, fluconazole, which targets *ERG11* in the fungal cell, was found. This implies that only a reduction in expression of genes in the pathway does not always affected azole resistance of the yeast. Moreover, the reduced activity of the ergosterol biosynthesis under the herein used condition, mainly the growth at 37°C, likely reflects the reduction of the need for sterols in membrane fluidity at high temperatures [62], which may explain the limited effectiveness of those drugs in febrile patients.

The herein presented results suggest that the comparison of the transcriptional profiles of different strains can be used to identify virulence genes or link specific genes with virulence. They support the concept that gene dosage or elevated transcripts can be linked to yeast phenotype for certain genetic loci, including virulence determinants.

Limitations of the approach we have taken for transcriptional profiling in this study include the following: The microarray available to us at the time of starting the work was based on a serotype D strain, which resulted in the fact that only 51% of the 70 mers probes of this array had ≥90% identity to the sequences of the strain R265 (Jason Stiajich, personal communication). This may mean that some of the differentially expressed genes were not detected. Only 50–60% of the genes on the microarray passed on our initial filtering (data not shown). However, based on the high sequence similarity between the coding regions of *C. neoformans* var. *neoformans* and *C. gattii* (see also the result section, Figure S1 and [28], [29], [31]) and the successful previous use of this mircroarray to study gene expression differences in the closely related variety *C. neoformans* var. *grubii* [63], it is a valuable tool to examine expression differences between the two VGII outbreak strains, compared in the current study. In addition to the array-based limitations, there may have been some mismatches in the hybridization results since the strains R265 and R272 are not genetically identical. However, according to MLST data based on 30 genetic loci and the mating type locus, there is less than 1% genetic difference between both strains [15]. Thus, the possibility of a mismatch between these strains is very low and should not have had a significant effect on the microarray results. Finally, the transcriptional profile of differentially genes was obtained using a single environmental condition and time point. Additional genetic determinants of virulence may be identified using different growth conditions.

In conclusion, substantial differences between the newly emerged and highly virulent VGIIa subtype (represented by strain R265) and the less virulent VGIIb subtype (represented by strain R272) of *C. gattii* have been identified by transcription profiling. Despite the genetic similarities between R265 and R272 [15], key differences in virulence (e.g. *LAC1, LAC2, CAS3* and *MPK1*), housekeeping (cell wall assembly, carbohydrate and lipid metabolism) and mitotic regulatory genes were identified. Further characterization of three putative virulence genes, *LAC1* (melanin synthesis), *CAS3* (capsule production) and *MPK1* (MAP kinase) revealed that over-expression of *LAC1* and *MPK1* was associated with an enhanced virulence phenotype *in vitro* and *in vivo,* whereas *CAS3* did not influence capsule size *in vitro*, but was associated with enhanced virulence *in vivo* (data not shown). A *C. gattii* serotype B microarray has now become available [33] and will allow gene expression in *C. gattii* strains to be studied directly. Nevertheless, the present study has highlighted strain-dependent differences in gene expression that correlate with high and lower virulence in a significant newly emerged pathogen. This work provides a basis for further investigations of mechanisms that regulate the virulence composite within pathogenic fungi.

## Materials and Methods

### Strains and Growth Conditions

The strain R265, representing the high virulent VGIIa genotype and strain R272 representing the low virulent VGIIb genotype causing the Vancouver Island cryptococcosis outbreak, identified in a previous study [15], were chosen for microarray comparison. The strains were retrieved from the freeze-dried culture collection of Molecular Mycology Research Laboratory, Westmead Hospital (Table 1). Each strain was maintained on Yeast Peptone Dextrose (YPD) agar (2% glucose, 2% peptone, 1% yeast extract, 2% agar) at 30°C and then subjected to the studies described below. In experiments using a specific number of colony forming units (CFU)/ml or optical density at 600 nm as starting points, *C. gattii* strains were initially grown overnight in YPD broth at 30°C and counted by hemocytometer for subsequent studies.

### DNA isolation

Genomic DNA was extracted and purified according to the method described previously with modification [64]. Briefly, 100 µl of the yeast pellet was frozen in a 1.5 ml microcentrifuge tube for 30 minutes at −20°C. 500 µl of lysis buffer (0.5% SDS, 1.4% NaCl, 0.73% EDTA, 0.2 M Tris-HCl) and 5 µl of 2-mercaptoethanol was added and incubated at 65°C for 1 hour. The mixture was vortexed vigorously. The DNA was purified by 25∶24∶1 phenol:chloroform:isoamyl alcohol and precipitated with isopropanol. The DNA pellet was washed once by 70% ethanol and resuspended in 100 µl of sterile double-distilled deionized water.

### mRNA isolation

In order to obtain mRNA from nutritionally stressed isolates, strains R265 and R272 were initially grown in YPD broth at 30°C for 24 h to saturation (∼10^8^ CFU/ml) and then pelleted. Cells were resuspended in equal volume of minimal medium (0.1% glucose YNB without amino acid and ammonium sulfate from Gibco, CA, USA) and incubated at 37°C for 1 h as previously published [36]. Cells were pelleted by centrifugation for RNA extraction. The yeast cell pellet was lyophilized and RNA was extracted using 0.5 µm glass beads vortexed in Trizol® reagent (Invitrogen, CA, USA) according to the manufacturer's instructions. The extracted RNA was further purified with the Qiagen® RNA mini kit (Qiagen, CA, USA) according to the manufacturer protocol.

### cDNA synthesis and labeling

In order to detect gene expressions by both R265 and R272 strains, a reference cDNA pool was prepared by mixing together an equal amount of RNA obtained from both strains after growth for 1 hr in eight different growth conditions based on previous publication [36]. The eight different growth conditions were as follows: I: YPD medium, 30°C; II: YPD medium, 37°C; III: YPD medium, 39°C; IV: complete YNB medium with 0.1% glucose [glucose limiting], 37°C; V: YNB without amino acid and ammonium sulfate with 0.1% glucose [nitrogen limiting], 37°C; VI: RPMI medium with 54 mM MOPS pH 7.3 incubated in room air, 37°C; VII: RPMI medium with 29 mM NaHCO_3_ and 25 mM MOPS pH 7.3 incubated in 5% CO_2_, 37°C; and VIII: RPMI medium with 54 mM MOPS and 0.75 M NaCl, 37°C. Fluorescent cDNAs were synthesized during reverse transcription of 10 µg of the total RNA reference pool by incorporating Cy3 dye (Amersham, NJ, USA) coupled to the amino-allyl group at the Duke Microarray Facility according to established protocols for custom spotted arrays (http://microarray.genome.duke.edu/). The cDNA from each strain was individually labeled with Cy5 dye as described above and competitively hybridized against the reference pool.

### Microarray hybridization

A whole-genome 70-mer oligonucleotide DNA microarray corresponding to each predicted open reading frame of the *C. neoformans* serotype D genome (strain JEC21) with additional genes of the *MAT* ***a*** and *MATα* loci from all serotypes was used. The composition of the microarray is deposited at MIAME (http://www.ebi.ac.uk) with the physicalArrayDesign name: DukeCND and the ArrayExpress accession: A-MEXP-1133. The microarray was designed and created by the Heitman Laboratory and the Duke Microarray Facility as described previously [63]. All hybridizations and data acquisitions were performed at the Duke Microarray Facility (http://microarray.genome.duke.edu/) according to their established protocols.

### Microarray data analysis

Microarray hybridizations were performed in triplicates for each strain. The comparison of the gene expression between the two strains was performed as follows: Microarray data were imported into the GeneSpring GX version 7.3.1 software (Agilent Technologies, CA, USA) and initial background subtractions were performed based on negative controls and intensity-dependent (Lowess) normalization using the replicate cross-gene error model implemented in the software. The background-subtracted data were then filtered based on confidence using the Benjamini Hochberg false-discovery rate and on the fold change using the cut-off point of a 2-fold difference implemented Table in the GeneSpring software package. Finally, the data were statistically analyzed by one-way ANOVA using parametric tests (not assuming variances equals) and p values of ≤0.05 to obtain the list of differentially expressed genes for further evaluation (Table S1). All genes with annotated function were blasted against the *Saccharomyces cerevisiae* genome database (http://www.yeastgenome.org/) and the non-redundant GenBank database to name each gene according to its homolog. Unknown or hypothetical genes were identified via translated BLAST searches (BLASTX) against GenBank. When this search resulted in significant homologies (*E* value<e^−50^), the closest gene homologues were assigned to those sequences. The protein sequences of the corresponding JEC21 genes were blasted against the draft R265 genome (*Cryptococcus gattii* serotype B Sequencing Project, Center for Genome Research, http://www.broadinstitute.org/annotation/genome/cryptococcus_neoformans_b/MultiHome.html) to obtained their supercontig positions (Table S2).

### Transcriptional analysis

To verify the obtained microarray results three genes (*LAC1, CAS3* and *MPK1*) from the up-regulated gene group, one gene (*STE3*) from the down-regulated group and one gene (*ETF1α*) with no expression differences were chosen to perform real time (RT-) PCR. The RT-PCR was performed as described previously [36] comparing to a house keeping gene, *ACT1*, using the -RTF and -RTR primers (Table 2). Induction levels (n-fold) were calculated by Microsoft Excel 2003 and compared directly between the two strains.

**Table 2:** Primers used in this study.

Primers	Primers sequences	Note
RLAC1RTF	5′ ACCTTCATGGCAACGAGTTC 3′	RNA quantification of *LAC1*
RLAC1RTR	5′ ACAACCACAGCCAACTTTCC 3′	RNA quantification of *LAC1*
RCAS3RTF2	5′ CTGACGCCTGTTGAGAACAATG 3′	RNA quantification of *CAS3*
RCAS3RTR2	5′ GTCAGCGTATGCTCTCCAGGTT 3′	RNA quantification of *CAS3*
RLAC2RTF	5′ ACCCTTTACTTCGTGTCGTCCA 3′	RNA quantification of *LAC2*
RLAC2RTR	5′ TCCACCCTCCATCCAGAAAGTA 3′	RNA quantification of *LAC2*
RMPK1RTF	5′ TGGATTTGTTGAGCAAGCTG 3′	RNA quantification of *MPK1*
RMPK1RTR	5′ TCCTTACAGGAGGCATGGAG 3′	RNA quantification of *MPK1*
RSTE3RTF	5′ TGGCTAGCGCCTCTCTTATC 3′	RNA quantification of *STE3*
RSTE3RTR	5′ CCGAAATAACTGCCTTCCAA 3′	RNA quantification of *STE3*
RETF1RTF	5′ GCTGCTAGGGTCGTATCTGG 3′	RNA quantification of *ETF1*
RETF1RTR	5′ ACATACAGCTCAGGGGCAAC 3′	RNA quantification of *ETF1*
RACT1RTF	5′ GTCCTACGAGCTTCCTGACG 3′	RNA quantification of *ACT1*
RACT1RTR	5′ GCAGACTCGAGACCAAGGAG 3′	RNA quantification of *ACT1*
NatF*	5′ CATGCAGGATTCGAGTGGCATG 3′	*NAT^r^* and *NEO^r^* cassette
NatR*	5′ GGAGCCATGAAGATCCTGAGGA 3′	*NAT^r^* and *NEO^r^* cassette
RLAC1F1	5′ CGACAGTCCTTGCTTCATCA 3′	*LAC1* construct amplification (mutation)
RLAC1F2	5′ TCCTCAGGATCTTCATGGCTCC GCTCGGCTGAAGATTCTTTG 3′	*LAC1* construct amplification (mutation)
RLAC1R1	5′ CTCACCTACGGGGAAGTTCA 3′	*LAC1* construct amplification (mutation)
RLAC1R2	5′ CATGCCACTCGAATCCTGCATG TTGACTATCCCCCGCATTAC 3′	*LAC1* construct amplification (mutation)
RCAS3F1	5′ AACTGGAAATGGTGGTGAGC 3′	*CAS3* construct amplification (mutation)
RCAS3F2	5′ TCCTCAGGATCTTCATGGCTCCC ATCTGGGTAATTGGGATGG 3′	*CAS3* construct amplification (mutation)
RCAS3R1	5′ TTTGGGCGATCGGTATAGAG 3′	*CAS3* construct amplification (mutation)
RCAS3R2	5′ CATGCCACTCGAATCCTGCATG CATGTAAAAATGGCGGAATG 3′	*CAS3* construct amplification (mutation)
RLAC1EF	5′ TGCCTTATGGGATTTTCAGG 3′	*LAC1* construct insertion confirmation
RLAC1ER	5′ GAGATTCCCGCTGATATTGC 3′	*LAC1* construct insertion confirmation
RCAS3EF	5′ CGGTACGCACTCCTTGAACT 3′	*CAS3* construct insertion confirmation
RCAS3ER	5′ GTGGAAGAGTGGACGTGGAT 3′	*CAS3* construct insertion confirmation
RLAC1FI	5′ CATCAACGGTCGTGTAGGTG 3′	*LAC1* insertion confirmation
RLAC1RI	5′ TGTACCATCGGTCTCCACAA 3′	*LAC1* insertion confirmation
RCAS3FI	5′ GCGGCCTATATGGATTCTCA 3′	*CAS3* insertion confirmation
RCAS3RI	5′ CGCATACTCACCAACCATTG 3′	*CAS3* insertion confirmation
RLAC1RCF	5′ TGTTCCCATCATCTTTCCTTGG 3′	*LAC1* construct amplification (complementation)
RLAC1RCR	5′ CATGCCACTCGAATCCTGCATG GATTTCGTTCGCTTGGTGTTTC 3′	*LAC1* construct amplification (complementation)
RCAS3RCF	5′ GGGGCGTAAGGAATGCTAAAAC 3′	*CAS3* construct amplification (complementation)
RCAS3RCR	5′ CATGCCACTCGAATCCTGCATG CGGTGGCTTTCCTGTTCTTATG 3′	*CAS3* construct amplification (complementation)
RMPK1F1	5′ CGGGGCACTATATGTCGAGT 3′	*MPK1* construct amplification (mutation)
RMPK1F2	5′ TCCTCAGGATCTTCATGGCTCC TCGAGTCTGTAACTGTTGTCGAA 3′	*MPK1* construct amplification (mutation)
RMPK1R1	5′ CATCATCCGGCTAGTGGTC 3′	*MPK1* construct amplification (mutation)
RMPK1R2	5′ CATGCCACTCGAATCCTGCATG GGGGTGTTGTCCATGGTATG 3′	*MPK1* construct amplification (mutation)
RMPK1EF	5′ CAGCCATCACCTCATCCTTT 3′	*MPK1* construct insertion confirmation
RMPK1ER	5′ GGCTTTTGGTCGTCTACTGC 3′	*MPK1* construct insertion confirmation
RMPK1FI	5′ CCCCTCGCGATACTAATTCA 3′	*MPK1* insertion confirmation
RMPK1RI	5′ TATGCGTGCGATCAGCTTAC 3′	*MPK1* insertion confirmation
RMPK1RCF	5′ GATCGGGGCACTATATGTCGAG 3′	*MPK1* construct amplification (complementation)
RMPK1RCR	5′ CATGCCACTCGAATCCTGCATG GGGTTGGATGAGGAAATGTTGA 3′	*MPK1* construct amplification (complementation)

*[56]

### Melanin synthesis test

Niger seed agar, caffeic acid agar and dopamine agar (0.5 mM dopamine) were used to test for melanin production [65]. Both strains were diluted and grown on those media at 30°C and 37°C. The ability to produce melanin was compared at 24 and 48 h by examining for the appearance of brown yeast colonies. Quantification of melanin production (laccase activity) was performed in triplicate according to a modified method described previously [37], using 0.1% glucose YNB without amino acid and ammonium chloride (Becton, Dickinson and Company®, MD, USA) with either 10 mM dopamine HCl (Sigma-Aldrich®, MD, USA) or 0.5 mM epinephrine (Sigma-Aldrich®, MD, USA).

### Capsule production

Capsule-inducing conditions were used. Briefly, each strain of *C. gattii* was grown overnight in YPD broth at 30°C. Yeast cells were then washed and grown to a cell density of 10^6^–10^7^ cells/ml using the following conditions: Dulbecco's modified Eagle's medium (DME) or RPMI with MOPS, HCO_3_, pH 7.3, in 5% CO_2_ at 37°C [66], [67]. Capsule size was quantified by light microscopy for at least 100 cells that had been cultured for 72 h and suspended in India ink using encapsulated to naked yeast size (cell wall to cell wall diameter) ratios.

### Cell wall integrity test

Dilutions of each strain were grown on YPD containing 0.2 M CaCl_2_, 7 mg/ml Calcofluor white, 5 mg/ml Congo red or 1 mg/ml Caffeine, which are known to cause perturbations of yeast cell wall [27], [68].

### Growth at 37°C test

Both strains were diluted and grown on either YPD or minimal media with 2% agar at 37°C.

### Mating test

Mating assays were carried out on V8 medium [69] (5% [vol/vol] V8 juice, 2 mM KH_2_PO_4_, 4% [wt/vol] agar). The ability to mate was tested against the VGII mating type **a** strains listed in Table 1. The molecular type and mating type of each strain were confirmed by *URA5* gene RFLP analysis [12] and mating type specific PCR [8]. Strains cultured on YPD agar for 2 days, were patched onto solid mating medium either alone or mixed with the mating type **a** strains. Plates were incubated at room temperature in the dark for 3 weeks and examined by light microscopy for filamentation and basidiospore formation. All experiments were done in duplicate using V8 media at pH 5 and 7.

### Fluconazole susceptibility

Antifungal susceptibility was determined using the CLSI broth microdilution method (M27-A2) [70].

### Characterization of *LAC1*, *CAS3* and *MPK1* genes

All genes were first identified in BLASTN searches against the *Cryptococcus gattii* strain R265 genome database (*Cryptococcus gattii* serotype B Sequencing Project, Center for Genome Research, http://www.broad.mit.edu) using homologous genes from the *Cryptococcus neoformans* strain JEC21. Deletion mutants and reconstituted strains were then created from strain R265 according to an established protocol [71]. Knockout constructs were created as previously described [8]. Briefly, the 5′ (primers -F1 and -R2) and 3′ (primers -F2 and -R1) flanking regions of the genes of interest were linked to the nourseothricin resistance cassette (primers NatF and NatR) by using overlapping PCR (Table 2). The resulting knockout constructs were biolistically transformed into strain R265 on YPD-sorbitol plates and then incubated at 30°C for 16 to 20 hr. Cells were resuspended in YPD broth, re-plated onto medium containing nourseothricin and further incubated for 3 to 4 days at 30°C. Mutants were selected based on nourseothricin resistance and confirmed by both PCR (primers -EF and -ER) and Southern hybridization methods. The mutants were reconstituted by transforming a reconstituted construct for each gene. The constructs were generated using overlapping PCR by linking each gene, amplified with primers -RCF and –RCR, with the neomycin resistance cassette (primers NatF and NatR) (Table 2). The reconstituted strains were then selected based on the G418 resistance and confirmed by conventional PCR using primers -FI and –RI, to check for construct insertion, and real time PCR with gene-specific primers, to test for recovery of wild-type transcription levels. (Primer sequences are listed in Table 2).

### Murine inhalation model

Balb/c mice were inoculated intranasally with 10^5^ cells in 50 µL PBS, divided and pipetted into each nostril [15]. Mice were housed and supplied with food and water *ad libitum*. Groups of 15 mice were infected using the wild-type strain R265, *mpk1Δ* mutant and *mpk1Δ::MPK1* complemented strain (approved by the Western Sydney Area Health Service Animal Ethics Committee, Protocol Number 5020.04). Quantitative cultures of lung and brain were used to compare tissue burdens between groups of mice sacrificed on day 4, 7 and 14 following inoculation. Lung and brain were homogenized, diluted and plated out onto YPD agar to determine the number of CFU/g of tissue, which was plotted against the time to determine the progression of the infection.

### Ethics Statement

Project Title: "Pathogenic fungi - Linkage between genotype and clinical disease? *C. neoformans/C. gattii* spp. complex as a model system. Approved by the Westmead Hospital Animal Ethics Committee, Protocol Number 5020.04.

### Statistical analyses

All statistical analyses including t-test and Chi-square test were conducted in SPSS version 15.0 (LEAD Technologies, USA). Data were statistically significant when p value<0.05.

## Supporting Information

Figure S1 Alignment of the coding regions of *LAC1* (A), *CAS3* (B) and *MPK1* (C) gene sequences of the *C. neoformans* var. *neoformans*, VNIV, strain JEC21 and the *C. gattii,* VGIIa, strain R265, revealing 84%, 84% and 92% similarities to the JEC21 genes, respectively.
(TIF)

Table S1 Complete list of all up- and down-regulated genes including open reading frames with known and unknown functions.
(DOC)

Table S2 List of gene functions of differentially expressed genes with transcription levels, which are at least 2-fold higher or lower in strain R265 vs. strain R272.
(DOC)
