# Supplementary figures and images for: Cryptococcus gattii Virulence Composite: Candidate Genes Revealed by Microarray Analysis of High and Less Virulent Vancouver Island Outbreak Strains
Source: PLoS One. 2011 Jan 13;6(1):e16076. doi: 10.1371/journal.pone.0016076 (PMC3020960; doi:10.1371/journal.pone.0016076)

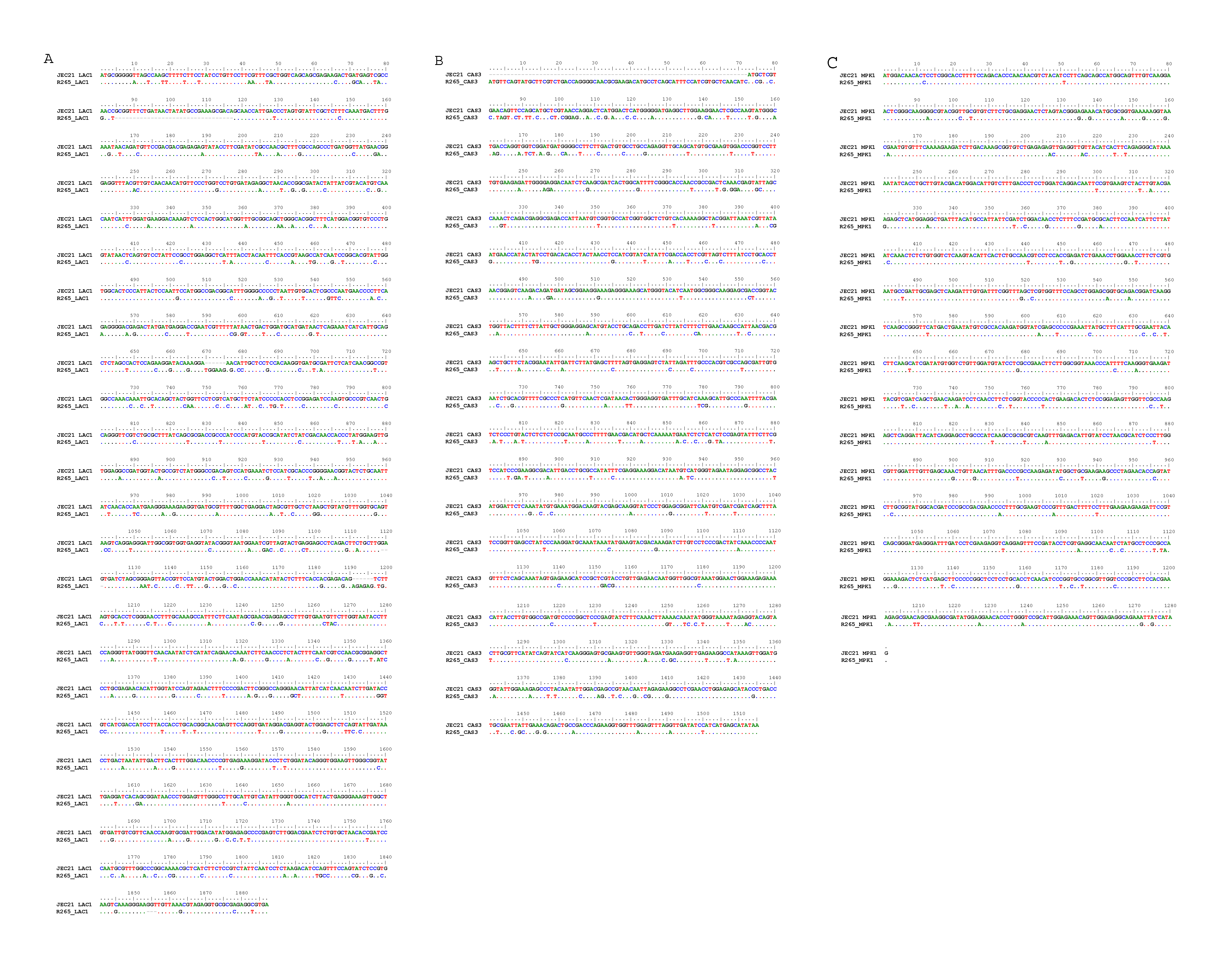

Supplement: Figure S1 — Alignment of the coding regions of LAC1 (A), CAS3 (B) and MPK1 (C) gene sequences of the C. neoformans var. neoformans, VNIV, strain JEC21 and the C. gattii, VGIIa, strain R265, revealing 84%, 84% and 92% similarities to the JEC21 genes, respectively. (TIF) [file pone.0016076.s001.tif]
