# Supplementary material for: Cryptococcus gattii Virulence Composite: Candidate Genes Revealed by Microarray Analysis of High and Less Virulent Vancouver Island Outbreak Strains
Source: PLoS One. 2011 Jan 13;6(1):e16076. doi: 10.1371/journal.pone.0016076 (PMC3020960; doi:10.1371/journal.pone.0016076)
Supplement: Table S1 — Complete list of all up- and down-regulated genes including open reading frames with known and unknown functions. (DOC) [file pone.0016076.s002.doc]

**Supplementary Table S1: Complete list of all up- and down-regulated genes including open reading frames with known and unknown functions.**

| Name of proteins | Genbank  Gene Symbol/  Locus Tag | Tigr  Accession No. | Gene expression  of R265/R272 |
| --- | --- | --- | --- |
| expressed protein | CNI00020 | 162.m02589 | 4.463 |
| MAP kinase putative | CNI00410 | 162.m02645 | 2.134 |
| thiamine pyrophosphokinase putative | CNI01170 | 162.m02670 | 2.12 |
| expressed protein | CNI00990 | 162.m02676 | 3.93 |
| polysaccharide synthase putative | CNI02290 | 162.m02779 | 3.455 |
| cell wall organization and biogenesis-related protein putative | CNI03080 | 162.m02845 | 2.517 |
| conserved hypothetical protein | CNI03260 | 162.m02853 | 2.273 |
| chitinase putative | CNI03860 | 162.m02909 | 0.469 |
| glyceraldehyde 3-phosphate dehydrogenase putative | CNI00320 | 162.m02964 | 3.078 |
| ferric reductase transmembrane component 2 precursor (ec 1.16.1.7) putative | CNI01220 | 162.m02981 | 2.166 |
| expressed protein | CNI02820 | 162.m03030 | 0.465 |
| acyl-CoA thioesterase putative | CNI04130 | 162.m03592 | 0.48 |
| Hypothetical protein |  | 1621.seq.137 | 0.465 |
| hypothetical protein | CND01780 | 163.m02846 | 0.445 |
| ferric-chelate reductase putative | CND00150 | 163.m03777 | 0.281 |
| aflatoxin efflux pump AFLT putative | CND00440 | 163.m03822 | 0.323 |
| nuclear cohesin complex protein putative | CND01530 | 163.m04709 | 2.284 |
| conserved hypothetical protein | CND01360 | 163.m04783 | 0.477 |
| expressed protein | CND02500 | 163.m06185 | 0.251 |
| hypothetical protein | CND02540 | 163.m06189 | 2.579 |
| expressed protein | CND05320 | 163.m06296 | 5.61 |
| hypothetical protein | CND05620 | 163.m06341 | 0.444 |
| conserved hypothetical protein | CND06160 | 163.m06415 | 23.49 |
| chitin deacetylase-like mannoprotein MP98 | CND03490 | 163.m06424 | 4.696 |
| alpha-16-mannosyltransferase putative | CND03380 | 163.m06429 | 2.032 |
| conserved hypothetical protein | CND04010 | 163.m06471 | 0.494 |
| expressed protein | CND04490 | 163.m06526 | 0.468 |
| cyclohydrolase putative | CNM00180 | 164.m01653 | 2.122 |
| conserved hypothetical protein | CNM00900 | 164.m02065 | 4.064 |
| conserved hypothetical protein | CNM02430 | 164.m02187 | 5.091 |
| acidic laccase putative | CNM02420 | 164.m02206 | 2.692 |
| hypothetical protein | CNM01490 | 164.m02800 | 0.387 |
| hypothetical protein |  | 1641.seq.128 | 0.498 |
| expressed protein | CNM01510 | 1641.seq.169 | 3.882 |
| hypothetical protein |  | 1642.seq.088 | 0.125 |
| Hypothetical protein |  | 1664.seq.126 | 0.433 |
| hypothetical protein | CNE00360 | 167.m03192 | 0.378 |
| endoplasmic reticulum protein putative | CNE00870 | 167.m03243 | 2.785 |
| conserved hypothetical protein | CNE03140 | 167.m03460 | 0.411 |
| cellulase putative | CNE03150 | 167.m03461 | 7.224 |
| ubiquitin conjugating enzyme putative | CNE03270 | 167.m03473 | 2.143 |
| expressed protein | CNE03900 | 167.m03535 | 0.332 |
| expressed protein | CNE04190 | 167.m03559 | 2.27 |
| phosphatidylinositol transporter putative | CNE04320 | 167.m03571 | 4.892 |
| glyoxal oxidase precursor putative | CNE05040 | 167.m03638 | 6.005 |
| conserved hypothetical protein | CNE00670 | 167.m05790 | 2.151 |
| conserved hypothetical protein | CNE00970 | 167.m05797 | 0.195 |
| cyclin-dependent protein kinase regulator putative | CNE04400 | 167.m05836 | 0.445 |
| chitin synthase putative | CNE03240 | 167.m05877 | 16.98 |
| hypothetical protein | CNE03230 | 167.m05878 | 0.476 |
| expressed protein | CNE02240 | 167.m05921 | 2.091 |
| copper chaperone putative | CNE01230 | 167.m05949 | 2.195 |
| hypothetical protein |  | 1672.seq.071 | 5.672 |
| hypothetical protein |  | 1682.seq.024 | 0.185 |
| Hypothetical protein |  | 1682.seq.176 | 0.367 |
| hypothetical protein |  | 1702.seq.064 | 2.151 |
| Hypothetical protein |  | 1702.seq.189 | 0.153 |
| glyoxal oxidase precursor putative | CNA03960 | 1703.seq.027 | 2.366 |
| conserved hypothetical protein | CNA06700 | 1704.seq.111 | 2.245 |
| expressed protein | CNB00960 | 1711.seq.003 | 0.162 |
| hypothetical protein | CNB02010 | 1712.seq.033 | 0.41 |
| Hypothetical protein |  | 1741.seq.039 | 0.443 |
| hypothetical protein |  | 1741.seq.102 | 4.874 |
| Hypothetical protein |  | 1744.seq.113 | 0.442 |
| hypothetical protein | CNN01670 | 1751.seq.089 | 0.409 |
| hypothetical protein | CNK00540 | 176.m02194 | 0.302 |
| oxidoreductase putative | CNK00680 | 176.m02209 | 0.418 |
| expressed protein | CNK01200 | 176.m02261 | 2.136 |
| serine/threonine-protein kinase (ec 2.7.1.-) putative | CNK01630 | 176.m02301 | 0.36 |
| conserved hypothetical protein | CNK01800 | 176.m02318 | 0.435 |
| hypothetical protein | CNK02060 | 176.m02344 | 0.418 |
| hypothetical protein | CNK02360 | 176.m02371 | 2.135 |
| signal transducer putative | CNK02520 | 176.m02385 | 0.284 |
| expressed protein | CNK02650 | 176.m02399 | 2.166 |
| hypothetical protein | CNK03310 | 176.m02457 | 0.353 |
| hypothetical protein | CNK03130 | 176.m02584 | 0.312 |
| expressed protein | CNK02560 | 1762.seq.096 | 2.027 |
| Hypothetical protein |  | 1762.seq.181 | 0.447 |
| phytase putative | CNG00030 | 177.m02859 | 115.6 |
| conserved hypothetical protein | CNG00700 | 177.m02923 | 0.105 |
| conserved hypothetical protein | CNG00880 | 177.m02938 | 3.295 |
| conserved hypothetical protein | CNG01200 | 177.m02969 | 0.142 |
| laccase (EC 1.10.3.2) precursor putative | CNG01240 | 177.m02973 | 13.24 |
| microtubule motor putative | CNG01340 | 177.m02984 | 0.356 |
| cytoplasm protein putative | CNG01720 | 177.m03021 | 2.467 |
| hypothetical protein | CNG01880 | 177.m03036 | 0.293 |
| hypothetical protein | CNG03500 | 177.m03190 | 2.183 |
| conserved hypothetical protein | CNG03970 | 177.m03236 | 0.355 |
| nuclear condensin complex protein putative | CNG04180 | 177.m03259 | 0.332 |
| 12 kda heat shock protein (glucose and lipid-regulated protein) putative | CNG04220 | 177.m03264 | 3.072 |
| conserved hypothetical protein | CNG04300 | 177.m03272 | 5.011 |
| UDP-glucosesterol transferase | CNG04310 | 177.m03273 | 2.23 |
| alpha-amylase putative | CNG04200 | 177.m03345 | 0.256 |
| expressed protein | CNG00370 | 177.m03363 | 2.48 |
| hypothetical protein | CNG00770 | 177.m03379 | 0.331 |
| diphenol oxidase putative | CNG01250 | 177.m03382 | 7.709 |
| hypothetical protein | CNG00010 | 1771.seq.001 | 3.272 |
| Hypothetical protein |  | 1773.seq.010 | 3.206 |
| Hypothetical protein |  | 1773.seq.017 | 0.337 |
| hypothetical protein | CNC00010 | 179.m00001 | 0.457 |
| conserved hypothetical protein | CNC00260 | 179.m00027 | 0.475 |
| expressed protein | CNC02080 | 179.m00194 | 0.108 |
| expressed protein | CNC02110 | 179.m00197 | 4.089 |
| expressed protein | CNC03380 | 179.m00298 | 2.652 |
| monooxygenase protein putative | CNC03590 | 179.m00313 | 2.613 |
| ER to Golgi transport-related protein putative | CNC03820 | 179.m00335 | 0.411 |
| phosphate transporter putative | CNC03960 | 179.m00344 | 4.373 |
| endoplasmic reticulum protein putative | CNC04620 | 179.m00396 | 2.431 |
| threonine aldolase putative | CNC04680 | 179.m00400 | 0.413 |
| conserved hypothetical protein | CNC06500 | 179.m00560 | 0.399 |
| expressed protein | CNC02050 | 179.m00660 | 2.511 |
| expressed protein | CNC03100 | 179.m00674 | 2.621 |
| potassium transport protein high-affinity putative | CNC04730 | 179.m00710 | 2.095 |
| expressed protein | CNC05430 | 179.m00718 | 0.351 |
| UDP-N-acetylglucosamine diphosphorylase putative | CNF01520 | 180.m00080 | 3.187 |
| hypothetical protein | CNF02490 | 180.m00115 | 4.459 |
| hypothetical protein | CNF03220 | 180.m00145 | 3.051 |
| conserved hypothetical protein | CNF03270 | 180.m00147 | 0.404 |
| hydroxymethylglutaryl-CoA reductase (NADPH) putative | CNF04830 | 180.m00238 | 0.408 |
| expressed protein | CNF01150 | 180.m00304 | 0.448 |
| chitin synthase-related | CNF01610 | 180.m00321 | 9.451 |
| conserved hypothetical protein | CNF02840 | 180.m00401 | 0.434 |
| Rho small monomeric GTPase putative | CNF04140 | 180.m00452 | 2.33 |
| pim1 protein (poly(a)+ RNA transport protein 2) putative | CNF04720 | 180.m00456 | 0.46 |
| hypothetical protein | CNA00210 | 181.m07808 | 2.162 |
| sterol 14-demethylase putative | CNA00300 | 181.m07816 | 0.474 |
| conserved hypothetical protein | CNA02630 | 181.m08023 | 2.399 |
| hypothetical protein | CNA02870 | 181.m08047 | 9.822 |
| hypothetical protein | CNA03140 | 181.m08074 | 2.083 |
| pyruvate dehydrogenase (acetyl-transferring) putative | CNA03850 | 181.m08143 | 2.217 |
| actin filament organization-related protein putative | CNA03860 | 181.m08144 | 2.371 |
| acetyl-CoA C-acyltransferase putative | CNA04700 | 181.m08224 | 2.558 |
| chitin synthase 6 (ec 2.4.1.16) putative | CNA05300 | 181.m08287 | 4.033 |
| expressed protein | CNA05700 | 181.m08321 | 40.75 |
| expressed protein | CNA06570 | 181.m08403 | 10.24 |
| conserved hypothetical protein | CNA06700 | 181.m08416 | 2.259 |
| alpha-mannosidase putative | CNA06750 | 181.m08419 | 2.513 |
| multidrug resistance protein 1 putative | CNA07730 | 181.m08516 | 0.25 |
| C-8 sterol isomerase putative | CNA08290 | 181.m08573 | 0.288 |
| conserved hypothetical protein | CNA04570 | 181.m08610 | 0.0743 |
| conserved expressed protein | CNA01170 | 181.m08704 | 2.306 |
| expressed protein | CNA01650 | 181.m08713 | 0.441 |
| glyoxal oxidase precursor putative | CNA03960 | 181.m08767 | 2.615 |
| conserved hypothetical protein | CNA05710 | 181.m08858 | 5.66 |
| conserved hypothetical protein | CNA06340 | 181.m08859 | 3.599 |
| expressed protein | CNA00080 | 181.m08863 | 2.865 |
| conserved hypothetical protein | CNA01600 | 181.m08868 | 0.428 |
| conserved hypothetical protein | CNA02400 | 181.m08869 | 0.216 |
| conserved hypothetical protein | CNN00300 | 183.m01610 | 0.458 |
| cytoplasm protein putative | CNN00410 | 183.m01620 | 3.543 |
| mitochondrion protein putative | CNN00830 | 183.m01655 | 0.452 |
| hypothetical protein | CNN01470 | 183.m01714 | 0.329 |
| cytoplasm protein putative | CNN02400 | 183.m01811 | 3.923 |
| glucan 13 beta-glucosidase protein putative | CNN00660 | 183.m01871 | 48.62 |
| conserved hypothetical protein | CNH00440 | 184.m04367 | 2.141 |
| conserved hypothetical protein | CNH00470 | 184.m04370 | 2.234 |
| Endoglucanase E-4 precursor (EC 3.2.1.4) putative | CNH00790 | 184.m04399 | 3.229 |
| conserved hypothetical protein | CNH01190 | 184.m04437 | 0.38 |
| hypothetical protein | CNH01410 | 184.m04458 | 3.578 |
| hypothetical protein | CNH01950 | 184.m04524 | 0.198 |
| syntaxin putative | CNH02560 | 184.m04593 | 2.207 |
| expressed protein | CNH02970 | 184.m04630 | 0.239 |
| v-SNARE putative | CNH03510 | 184.m04685 | 2.603 |
| heat shock protein putative | CNL04260 | 184.m04755 | 0.429 |
| DNA helicase putative | CNL04770 | 184.m04801 | 0.493 |
| exo-beta-13-glucanase | CNL04840 | 184.m04807 | 17.11 |
| expressed protein | CNL05810 | 184.m04898 | 2.477 |
| mitotic chromosome condensation-related protein putative | CNL06800 | 184.m04990 | 0.428 |
| exo-beta-13-glucanase | CNL04840 | 184.m05026 | 18.27 |
| cell wall organization and biogenesis-related protein putative | CNL05400 | 184.m05031 | 2.064 |
| expressed protein | CNL05090 | 184.m05170 | 0.417 |
| hypothetical protein | CNL06660 | 184.m05205 | 0.45 |
| response to drug-related protein putative | CNJ00600 | 185.m02405 | 0.484 |
| hypothetical protein | CNJ01860 | 185.m02531 | 4.306 |
| conserved hypothetical protein | CNJ03020 | 185.m02657 | 2.203 |
| expressed protein | CNJ03130 | 185.m02665 | 0.414 |
| hypothetical protein | CNJ03260 | 185.m02681 | 2.149 |
| expressed protein | CNJ03390 | 185.m02695 | 0.458 |
| conserved hypothetical protein | CNJ01130 | 185.m02739 | 6.709 |
| conserved hypothetical protein | CNJ02140 | 185.m02754 | 2.345 |
| conserved hypothetical protein | CNJ02150 | 185.m02769 | 0.492 |
| expressed protein | CNB00170 | 186.m03427 | 2.278 |
| expressed protein | CNB00910 | 186.m03503 | 2.067 |
| multidrug resistance protein 1 putative | CNB01030 | 186.m03521 | 0.384 |
| CAP64 gene product - related | CNB01440 | 186.m03566 | 8.238 |
| tubulin binding protein putative | CNB01560 | 186.m03577 | 0.267 |
| acyl-CoA dehydrogenase long-chain specific precursor putative | CNB01650 | 186.m03588 | 3.342 |
| hypothetical protein | CNB01680 | 186.m03592 | 0.402 |
| microtubule motor putative | CNB02110 | 186.m03639 | 0.484 |
| expressed protein | CNB02160 | 186.m03645 | 0.442 |
| conserved hypothetical protein | CNB02330 | 186.m03661 | 3.218 |
| cytoskeletal protein binding protein putative | CNB02580 | 186.m03682 | 0.22 |
| hypothetical protein | CNB03300 | 186.m03765 | 4.141 |
| carbon utilization by utilization of organic compounds-related protein putative | CNB04080 | 186.m03845 | 2.437 |
| conserved hypothetical protein | CNB04790 | 186.m03910 | 0.478 |
| hypothetical protein | CNB05700 | 186.m04014 | 0.415 |
| STE3 alpha (serotype B) |  |  | 0.371 |
| STE3 alpha (serotype C) |  |  | 0.226 |
| ETF1 a (serotype B) |  |  | 0.249 |
| ETF1 a (serotype C) |  |  | 0.13 |
