# Supplementary material for: Cryptococcus gattii Virulence Composite: Candidate Genes Revealed by Microarray Analysis of High and Less Virulent Vancouver Island Outbreak Strains
Source: PLoS One. 2011 Jan 13;6(1):e16076. doi: 10.1371/journal.pone.0016076 (PMC3020960; doi:10.1371/journal.pone.0016076)
Supplement: Table S2 — List of gene functions of differentially expressed genes with transcription levels, which are at least 2-fold higher or lower in strain R265 vs. strain R272. (DOC) [file pone.0016076.s003.doc]

**Table S2:** List of gene functions of differentially expressed genes with transcription levels, which are at least 2-fold higher or lower in strain R265 vs. strain R272.

| **Annotation** | **Fold change**  **of R265/R272**  **(RT PCR)** | **GenBank**  **Gene Symbol/**  **Locus Tag** | **Supercontig Numbers of R265 Genome** |
| --- | --- | --- | --- |
| **Genes with known roles in virulence composite** |  |  |  |
| Laccase precursor (*LAC1*)* | 13.24 (34.3)# | CNG01240 | 5 |
| CAP64 gene product related (*CAS3*)* | 8.238 (20.6)# | CNB01440 | 1 |
| Diphenol oxidase (*LAC2*)* | 7.709 (2.5)# | CNG01250 | 5 |
| MAP kinase (*MPK1*)* | 2.134 (2.15)# | CNI00410 | 6 |
| **Carbohydrate Metabolism** |  |  |  |
| Glucose oxidase | 4.064 | CNM00900 | 13 |
| Glyceraldehyde-3-phosphate dehydrogenase | 3.078 | CNI00320 | 6 |
| Alpha-D-mannosidase | 2.513 | CNA06750 | 3 |
| Carbon utilization by utilization of organic compounds-related protein | 2.437 | CNB04080 | 16 |
| Pyruvate dehydrogenase, acetyl transferrring | 2.217 | CNA03850 | 3 |
| Cyclohydrolase | 2.122 | CNM00180 | 13 |
| Thiamine pyrophosphokinase | 2.12 | CNI01170 | 6 |
| Alpha amylase | 0.256 | CNG04200 | 5 |
| **Cell wall assembly** |  |  |  |
| Glucan 1,3 beta-glucosidase | 48.62 | CNN00660 | 14 |
| Beta-1,6 glucan synthetase | 23.49 | CND06160 | 18 |
| Exo-beta-1,3-glucanase | 17.11 | CNL04840 | 12 |
| Chitin synthase 7 (*CHS7*)* | 16.98 | CNE03240 | 15 |
| Chitin synthase 5 (*CHS5*)* | 9.451 | CNF01610 | 4 |
| Cellulase | 7.224 | CNE03150 | 15 |
| Chitin deacetylase-like mannoprotein MP98 | 4.696 | CND03490 | 2 |
| Chitin synthase 6 (*CHS6*)* | 4.033 | CNA05300 | 3 |
| Polysaccharide synthase | 3.455 | CNI02290 | 6 |
| Endoglucanase E-4 precursor | 3.229 | CNH00790 | 11 |
| UDP-N-acetylglucosamine diphosphorylase putative | 3.187 | CNF01520 | 4 |
| Cytoplasm protein | 2.467 | CNG01720 | 5 |
| Chitinase | 0.469 | CNI03860 | 6 |
| **Transporters** |  |  |  |
| Phytase | 115.6 | CNG00030 | 5 |
| Phosphatidylinosterol transporter | 4.892 | CNE04320 | 15 |
| Phosphate transporter | 4.373 | CNC03960 | 8 |
| Iron transporter | 5.091 | CNM02430 | 13 |
| Acidic laccase | 2.692 | CNM02420 | 13 |
| Copper chaperone (*ATX1*)* | 2.195 | CNE01230 | 9 |
| Ferric reductase transmembrane component 2 precursor | 2.166 | CNI01220 | 6 |
| Ferric chelate reductase | 0.281 | CND00150 | 21 |
| **Lipid metabolism** |  |  |  |
| 3-hydroxybutyryl-CoA dehydrogenase | 5.011 | CNG04300 | 5 |
| Phytanoyl-CoA dioxygenase family | 4.463 | CNI00020 | 11 |
| Acyl-CoA dehydrogenase long-chain specific precursor | 3.342 | CNB01650 | 1 |
| Acetyl-CoA C acyltransferase | 2.558 | CNA04700 | 3 |
| UDP-glucose:sterol transferase | 2.23 | CNG04310 | 5 |
| Acyl-COA thioesterase | 0.48 | CNI04130 | 6 |
| oxidoreductase | 0.418 | CNK00680 | 7 |
| **Ergosterol biosynthesis** |  |  |  |
| Sterol 14 alpha-demethylase (*ERG11*)* | 0.474 | CNA00300 | 1 |
| hydroxymethylglutaryl-CoA reductase, NADPH | 0.408 | CNF04830 | 4 |
| C-8 sterol isomerase | 0.288 | CNA08290 | 3 |
| **Lignin degradation** |  |  |  |
| Glyoxal oxidase | 6.005 | CNE05040 | 15 |
| Glyoxal oxidase | 2.615 | CNA03960 | 3 |
| **Mitosis regulation** |  |  |  |
| Rho_small_monomeric_GTPase | 2.33 | CNF04140 | 4 |
| Nuclear cohesin complex | 2.284 | CND01530 | 2 |
| DNA helicase | 0.493 | CNL04770 | 12 |
| Microtubule motor | 0.484 | CNB02110 | 1 |
| Pim1 protein | 0.46 | CNF04720 | 4 |
| Cyclin-dependent protein kinase regulator | 0.445 | CNE04400 | 15 |
| Histone deacetylase | 0.434 | CNF02840 | 4 |
| Mitotic chromosome condensation-related protein | 0.428 | CNL06800 | 12 |
| Kinesin | 0.428 | CNA01600 | 1 |
| Microtubule motor | 0.356 | CNG01340 | 5 |
| Cytoskeletal protein binding protein | 0.22 | CNB02580 | 1 |
| Structural maintenance of chromosome 4 (*SMC4*)* | 0.195 | CNE00970 | 9 |
| **Drug Resistance** |  |  |  |
| Response to drug-related protein | 0.484 | CNJ00600 | 2 |
| Multidrug resistance protein 1 | 0.384 | CNB01030 | 1 |
| Aflatoxin efflux pump | 0.323 | CND00440 | 21 |
| Multidrug resistance protein 1 (*MDR1*)* | 0.25 | CNA07730 | 3 |
| **Stress response proteins** |  |  |  |
| 12 kda heat shock protein | 3.072 | CNG04220 | 5 |
| Ubiquitin conjugating enzyme | 2.143 | CNE03270 | 15 |
| Heat shock protein | 0.429 | CNL04260 | 12 |
| **Membrane protein** |  |  |  |
| v-SNARE | 2.603 | CNH03510 | 17 |
| Syntaxin | 2.207 | CNH02560 | 11 |
| **Signal transduction** |  |  |  |
| serine/threonine protein kinase | 0.36 | CNK01630 | 7 |
| signal transducer | 0.284 | CNK02520 | 7 |
| **Others** |  |  |  |
| Endoplasmic reticulum protein | 2.431 | CNC04620 | 8 |
| Mitrochodrion protein | 0.452 | CNN00830 | 14 |
| Threonine aldolase | 0.413 | CNC04680 | 8 |
| ER to Golgi transport-related protein | 0.411 | CNC03820 | 8 |
| Phosphatidylinositol 4,5-bisphosphate 5-phosphatase | 0.399 | CNC06500 | 10 |
| Synaptojanin binding protein | 0.388 | N |  |
| a-factor pheromone receptor (*STE3α*=*CPRα*)* | 0.371 (0.02) # | AY710430 | 18 |
| Polyamine transport protein | 0.302 | CNK00540 | 7 |

N = No Genbank Gene Symbol/Locus Tag No., * = Gene name was given to the gene according to previous studies in *C. neoformans* except *CHS5* and *SMC4* which were given e value = 0 in *Ustilago maydis* and *Saccharomyces cerevisiae* respectively., # Genes for which the level of transcription was confirmed by real time PCR (The control (higher-expressed) genes, *ETF1α*(1.15), is not on the list)
